# Supplementary material for: Accounting for cis-regulatory constraint prioritizes genes likely to affect species-specific traits
Source: Genome Biol. 2023 Jan 19;24:11. doi: 10.1186/s13059-023-02846-8 (PMC9850818; doi:10.1186/s13059-023-02846-8)
Supplement: Supplementary file 1 — Additional file 1: Figure S1. Relationship between the variance of the GTEx ASE distribution and the probability of Haploinsufficiency score. Figure S2. Relationship between population ASE variance and pHI when controlling for expression. Figure S3. Effect of population ASE sample size on correlation with pHI. Figure S4. Visualization of changes in gene ranking after incorporation of population ASE constraint. Figure S5. Exploration of changes in CUX1 expression. Figure S6. Changes in EDNRB expression are human derived. Figure S7. Many gene expression changes have the same direction in hybrid and parental organoids. Figure S8. Changes in many gluconeogenesis genes are human-derived. [file 13059_2023_2846_MOESM1_ESM.docx]

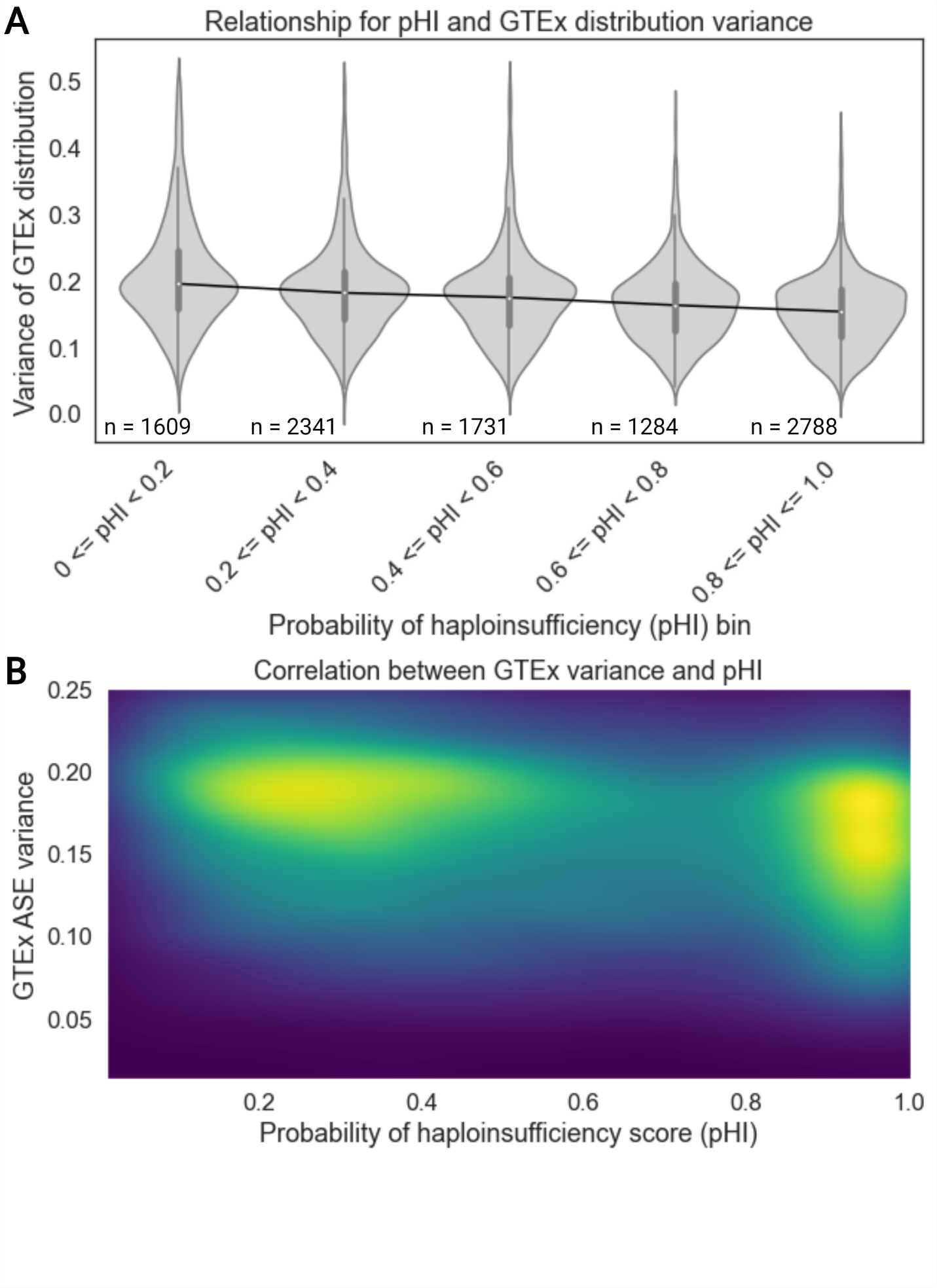


**Figure S1 Relationship between the variance of the GTEx ASE distribution and the probability of Haploinsufficiency score:** **A)** Shows the relationship between the variance of the GTEx ASE distribution and the probability of Haploinsufficiency score (a measure of constraint on gene expression) for each gene tested in this manuscript. As the probability of Haploinsufficiency increases, the variance of the GTEx distribution decreases. The Spearman correlation is -0.28 with p < 10^-170^. This correlation was computed on the raw pHI and ASE variance values that were not binned. We binned pHI scores to more accurately represent the strength of the trend we observed as the non-uniform distribution of pHI scores combined with the nearly normal distribution of GTEx ASE variances makes it difficult to plot the correlation in an informative manner without binning. The plotted line connects the medians of each distribution and the number of genes in each bin is represented by n. We restricted to only genes with GTEx ASE variance less than or equal to 0.5 for visualization purposes in this plot. **B)** The same data as in A but plotted as a density plot. To aid visualization as a density plot, we removed the genes with GTEx ASE variance less than 0.25. This was done only in this plot. As the pHI score increases, the density shifts toward lower ASE variance.


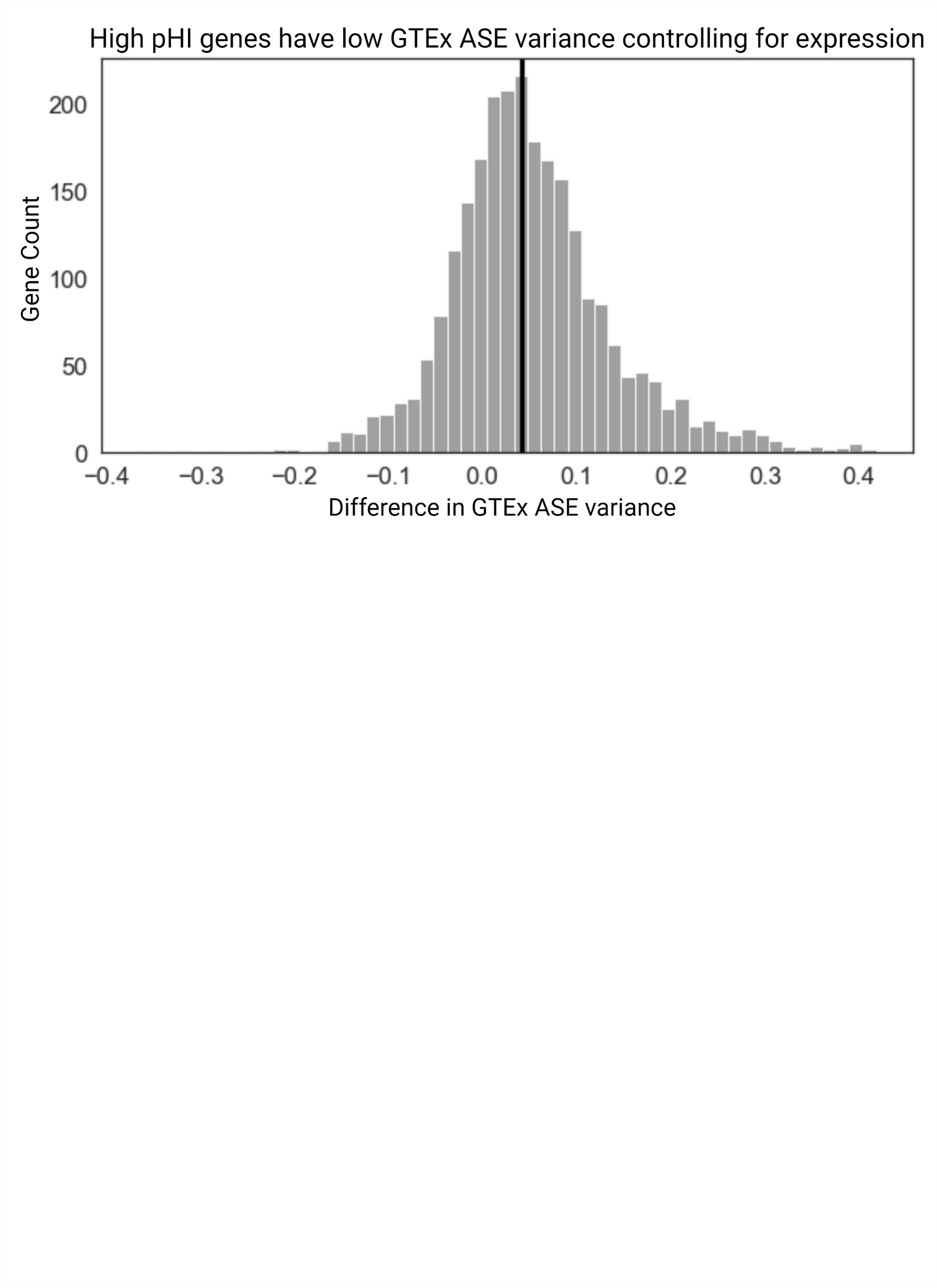


**Figure S2 Relationship between population ASE variance and pHI when controlling for expression:** Histogram of differences in ASE variance between genes with high and low pHI matched for expression. Low pHI genes generally have higher ASE variance even when controlling for expression which is reflected in the right shift of the distribution. The vertical black line is the median of the distribution.


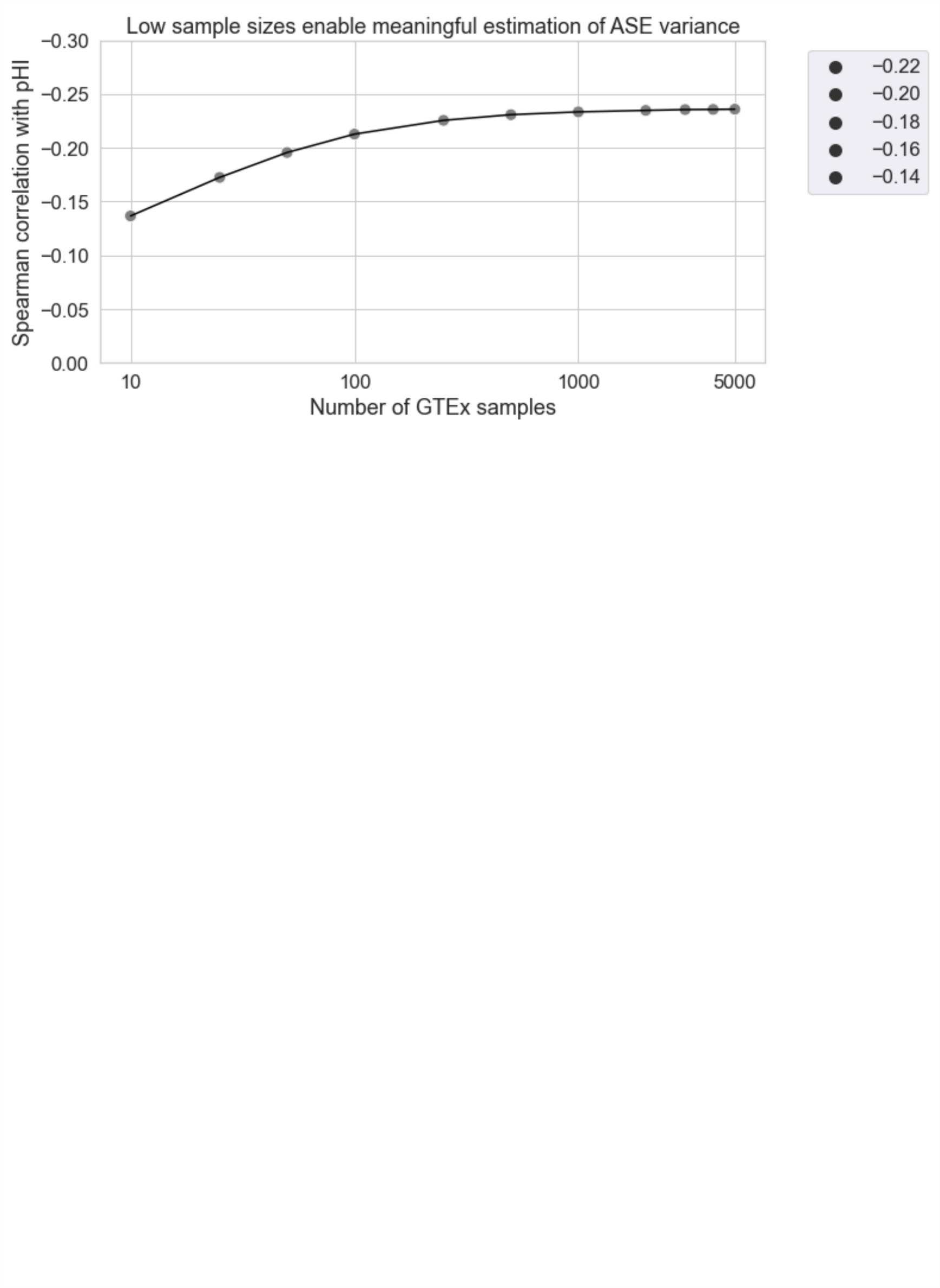


**Figure S3 Effect of population ASE sample size on correlation with pHI:** Plot showing the average correlation with pHI across 100 independently down-sampled sets of samples from GTEx. The black line connects the center of adjacent points and the size of the points reflects the sample size. The x-axis is on a log_10_ scale. The correlation with pHI saturates around 1000 samples and is still highly significant even with only 10 samples. Correlations are all slightly less than the correlation with the full GTEx dataset as only genes with greater than or equal to 5000 samples were included for the calculation of these correlations.


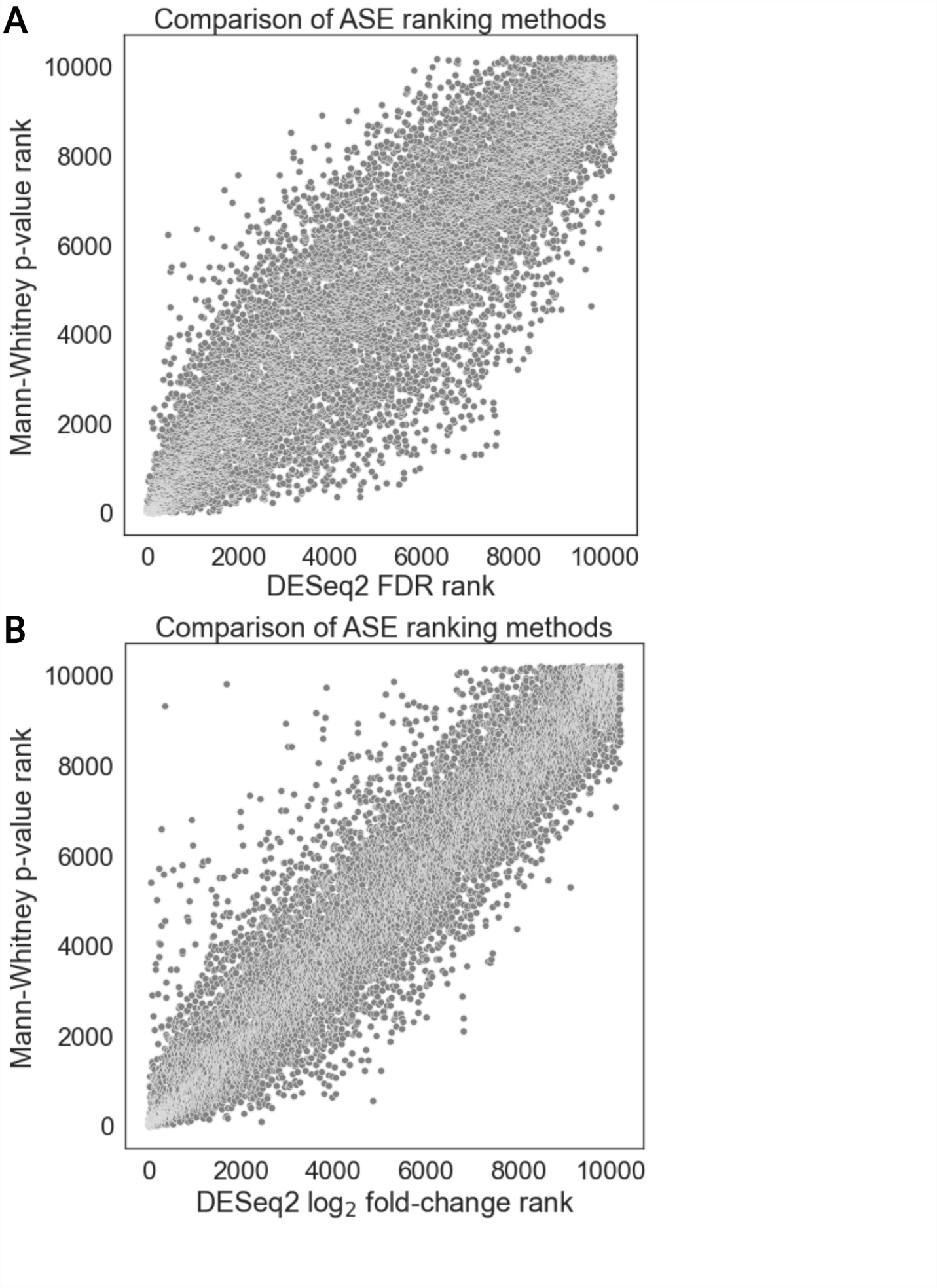


**Figure S4 Visualization of changes in gene ranking after incorporation of population ASE constraint: A)** Scatterplot of the DESeq2 FDR rankings and the Mann-Whitney p-value rankings which incorporate constraint on ASE. **B)** The same as A but ranking by DESeq2 log_2_ fold-change instead of DESeq2 FDR. Incorporating constraint on ASE impacts the rankings in both cases.


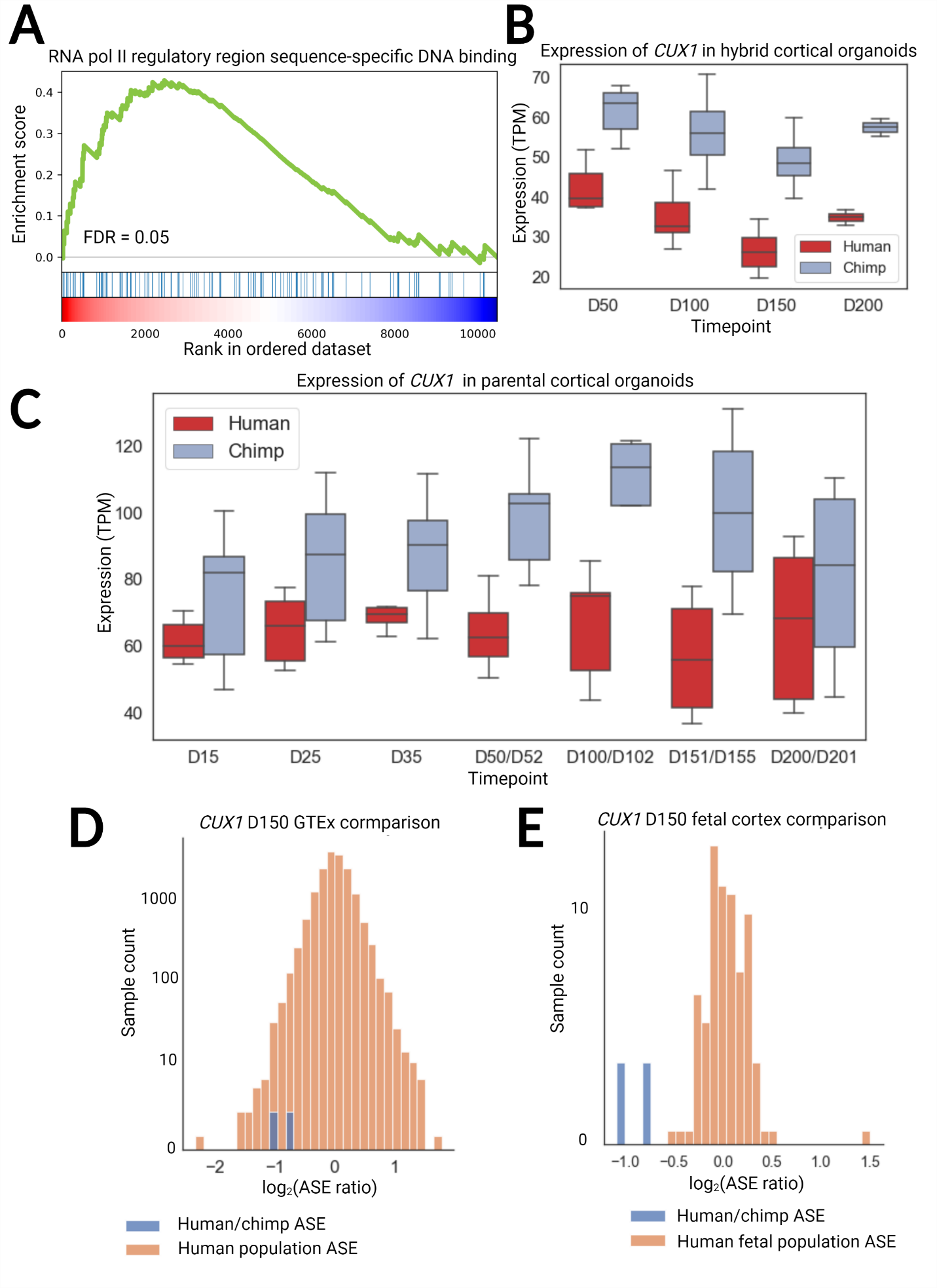


**Figure S5 Exploration of changes in *CUX1* expression:** **A)** Summary of RNA polymerase II regulatory region sequence-specific DNA binding. Each blue line represents a gene in the gene set and the green curve is the cumulative enrichment score. Genes in the gene set are enriched at the top of the list. **B)** Expression of *CUX1* in human-chimpanzee hybrid cortical organoids. Expression is shown in transcripts per million (TPM). Expression from the chimpanzee allele is consistently higher than expression from the human allele. **C)** Expression of *CUX1* in human and chimpanzee parental cortical organoids. Expression is shown in transcripts per million (TPM). **D)** Comparison of the GTEx ASE distribution for *CUX1* to the human-chimpanzee hybrid ASE distribution. Human-chimpanzee hybrid ASE is more extreme than ASE in 97.5% of GTEx samples. **E)** Comparison of the fetal cortex ASE distribution for *CUX1* to the human-chimpanzee ASE distribution. The human-chimpanzee hybrid ASE values lie well outside the human fetal cortex distribution.


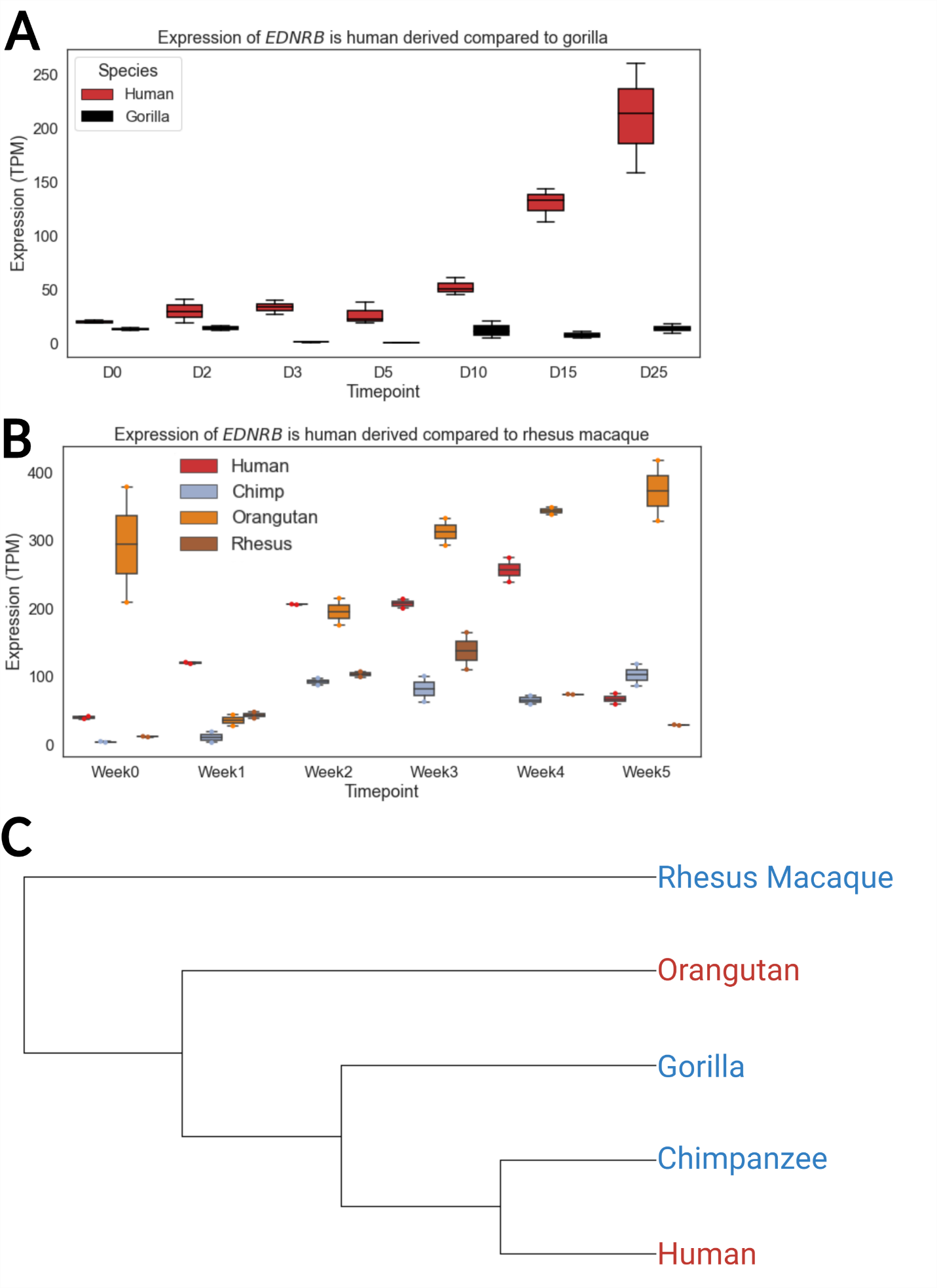
 **Figure S6 Changes in *EDNRB* expression are human derived:** **A)** Comparison of *EDNRB* expression between early-stage human and gorilla cerebral organoids. Human expression is considerably higher than gorilla expression across timepoints indicating that *EDRNB* is upregulated in the human lineage (as opposed to downregulated in the chimpanzee lineage). Data are from Benito-Kwiecinski *et al* [53]. **B)** Comparison of *EDNRB* expression between early-stage human, chimpanzee, orangutan, and rhesus macaque cerebral organoids. Human expression is considerably higher than rhesus macaque expression across timepoints. However, orangutan *EDNRB* expression is high as well, indicating an independent increase in expression in the orangutan lineage. Data are from Field *et al* [51]. **C)** Phylogenetic tree of old-world primates. Red text indicates a high *EDNRB* expression and blue text indicates low expression. Notably, as gorillas are more closely related to humans than organgutans this implies that the most parsimonious explanation for the data is that the last common ancestor of gorillas, chimpanzees, and humans had low *EDNRB* expression and that there was an increase in the human lineage.


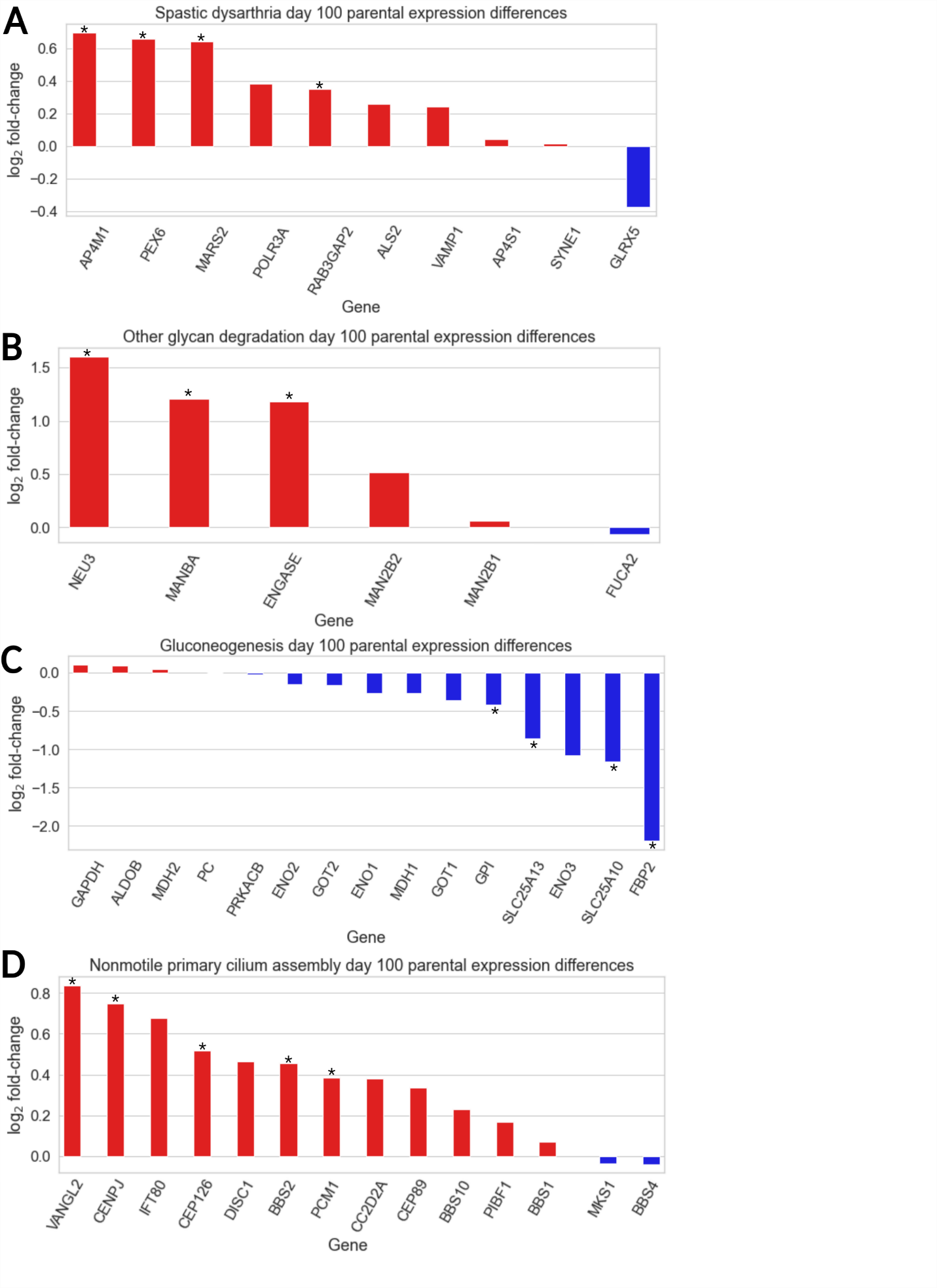


**Figure S7 Many gene expression changes have the same direction in hybrid and parental organoids: A)** Barplot showing the log_2_ fold-changes of genes driving the *Spastic dysarthria* enrichment comparing parental human and chimpanzee cortical organoids at day 100 of differentiation. Asterisks indicate a significant difference for that gene at an FDR cutoff of 0.1. **B)** Same as in A but for genes driving the *Other glycan degradation* enrichment. **C)** Same as in A, but for genes driving the *Gluconeogenesis* enrichment. **D)** Same as in A, but for genes driving the *Nonmotile primary cilium enrichment* as well as the single gene with chimpanzee-biased ASE (*MKS1*).


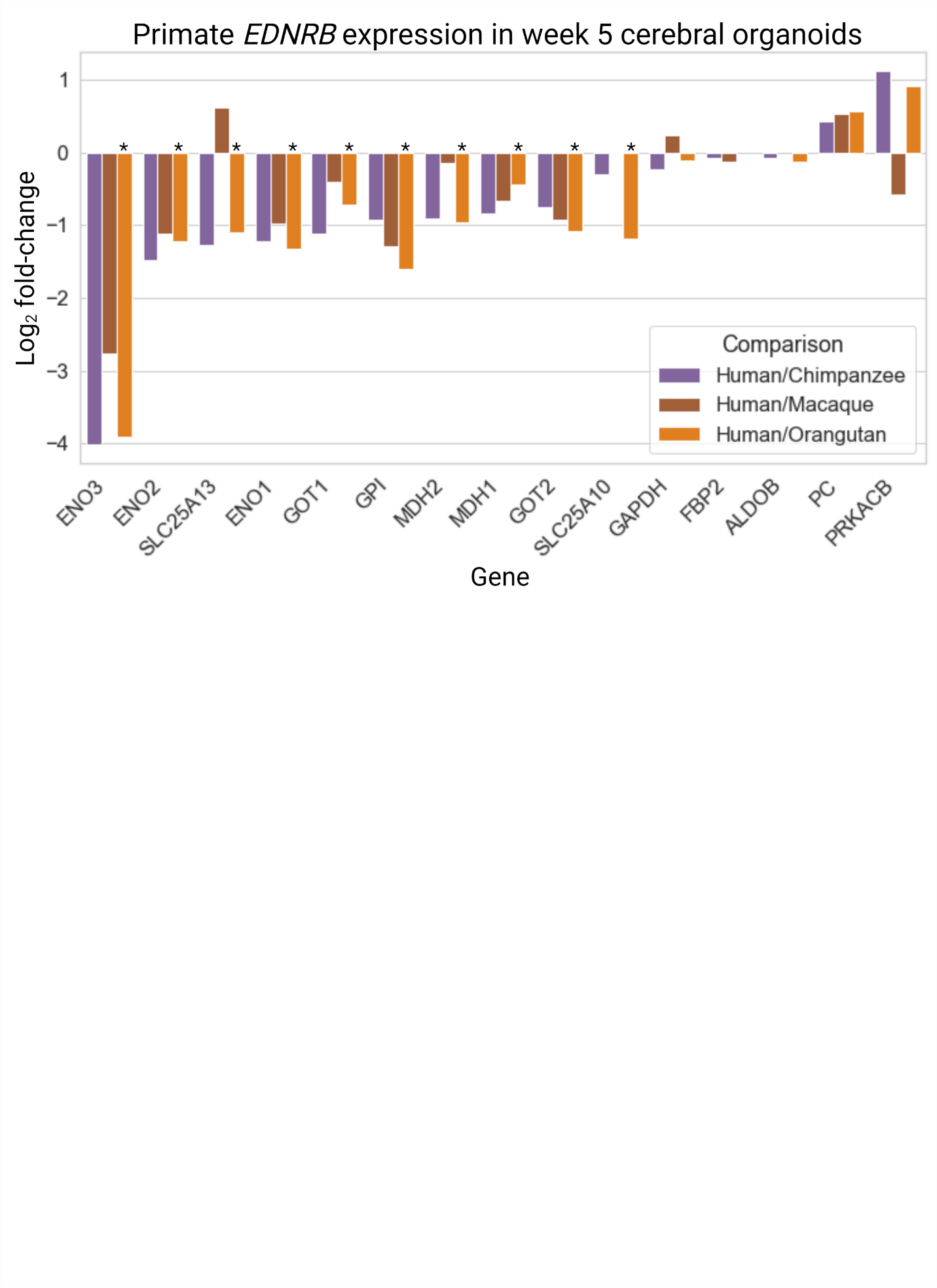


**Figure S8 Changes in many gluconeogenesis genes are human-derived:** Barplot showing that many changes in the expression of gluconeogenesis genes are human-derived and occur in parental organoids. Data are from Week 5 cerebral organoids in the manuscript by Field *et al* [51]. Asterisks indicate genes for which the human-orangutan difference is significant at an FDR cutoff of 0.1 and with lower expression in human. Genes whose down-regulation is not human-derived generally show insignificant differences in expression between humans and chimpanzees potentially indicating compensatory trans-acting genetic changes.
