# Supplementary material for: Accounting for cis-regulatory constraint prioritizes genes likely to affect species-specific traits
Source: Genome Biol. 2023 Jan 19;24:11. doi: 10.1186/s13059-023-02846-8 (PMC9850818; doi:10.1186/s13059-023-02846-8)
Supplement: Supplementary file 3 — Additional file 3. Review history. [file 13059_2023_2846_MOESM3_ESM.docx]

Review History

**First round of review**

**Reviewer 1**

**Were you able to assess all statistics in the manuscript, including the appropriateness of statistical tests used? No**

**Were you able to directly test the methods? No**

**Comments to author:**

The manuscript by Starr et al advocates for the wider use of existing public data of within-species allele specific expression variation to calibrate inter-species differences and identify those that are likely to be of evolutionary interest from those that aren't. This is an approach that has long been theoretically advocated for in DE testing, as the references in line 74 prove, but hasn't been actively implemented in ASE testing. The manuscript has two parts, first laying out the idea behind the new method and its implementation, and then combines it with the sign test in an attempt to identify instances of concerted changes in gene expression between humans and chimpanzees that span multiple genes.

I do like the idea in principle, and I think it's a nice complement to existing challenges in inter-species evaluation of ASE differences, although suitable datasets will be hard to come by, sadly! Having said that, I also think that there's a lot of ideas here about the validity of sorting genes by p-value and then by p-value again that need more thoughtful exploring from a statistical standpoint, to validate the method; I write this review from the perspective of a potential user, which is what I am, not a statistician. I do highlight some places where the authors can begin addressing that in my more specific comments, as follows:

Line 108: "We then use those p-values to separately rank genes with DESeq2 false discovery rate (FDR) less than or equal to 0.1 and greater than 0.1 such that genes with FDR less than or equal to 0.1 are always ranked higher than genes with FDR greater than 0.1." Isn't this sentence backwards? I presume the authors mean that they use the FDR to re-sort the list of p-values from their M-WU test such that MW-U p 0.15 and DESeq FDR 0.09 comes before MW-U p 0.00000000232 and DESeq FDR 0.101? This is also what is suggested by lines 419 onwards.

Line 119: How far down the evolutionary tree do the authors think human data could be use to inform inter-species ASE comparisons?

Line 132: It's not clear to me that haploinsufficiency and constraint on ASE would go hand in hand. The assumption seems sensible on first pass, but simply considering ASE doesn't actually control for differences in expression, as they themselves state, and despite the correlation in the text it's hard to see the trend given that the authors have plotted the data as boxplots instead of eg a scatter plot or a 2D density plot or a hex bin plot would be preferable. It's also not clear why the data is binned in the plot for pHI but, presumably, not for the calculating of the correlation reported in text.

Line 158: The fetal cortex ASE dataset is built from a max of 160 samples (as far as I can tell from looking at the reference - 85 neural progenitors and 74 differentiated progeny, although methods state 235 samples - where are the extra ones coming from?), a *lot* fewer than the GTEx dataset. Having said that, a visual count of the bar heights suggests that figure S2E included only ~70-80 data points. Are the CUX1 ASE values also outliers if GTEx is downsampled to the same number of samples? More broadly, and thinking of moving beyond human/ape comparisons - do the authors have any thoughts as to what is the minimum sample size needed to build a robust within-species ASE dataset? Does this vary between genes, eg, power estimates?

Line 172: One thing missing for me from these analyses, and this section, is the actual rank correlation between rankings between those two methods, and the actual plotting of rank changes. By using the method in figure 1A, the naive experimenter would sort their genes by their between species ASE p-value only before GSEA, whereas with this method the ranking depends on two different metrics now - the difference in within and between species ASE *and* DE testing results, which if I understood line 108 correctly, will rearrange the gene list such that all DE genes are ranked higher than all non DE genes, but not change rankings within those groups?

So, how significant an impact does this process have, on average? What's the rank correlation between looking only at ASE and looking at ASE and DE? How much of a difference does not controlling for DE have? In figure 3, the authors skip straight to showing that genes that change rank a lot between the two approaches are enriched for neural development terms when considering data from hybric cortical spheroids. I note that according to line 428, this comparison is not directly performed at any point? Instead the authors compare the GO results between using only DE FDR to rank genes, and their combined method, rather than ASE pval, which seems far more relevant to the question (since DE methods actively control for intra-group variance using empirical Bayes approaches etc, but the M-WU approach does not, since each test is independent of all other tests and does not borrow data across genes or perform any sort of shrinkage)

Box 1: The format of the manuscript is confusing - results are shunted to boxes, where they're freely mixed with some fairly speculative discussion. I presume this is a carryover of formatting for a previous journal, but if not, I urge the authors to reconsider it, I found it frustrating. The CUX1 box in particular segues into what's clearly discussion after a brief walk through the results, and makes some fairly large speculative leaps with regards to neoteny that don't really belong in the middle of the results section. If the authors want to look more into it, there are brain organoid datasets out there from human and chimp.

Line 250: "EDN1 had the strongest chimpanzee bias (mean log 2fold-change = -0.92, FDR < 0.025 at Day 150, Fig 4B)." I think there's something weird going on with figure 4B. EDN1 does indeed have a log FC of ~ -0.8 (could well be 0.92 but there's no tickmarks on the axes, please add those back) but the DE testing FDR-adjusted p value is 0.1 according to the y axis, not < 0.025 (-log10 (0.1) = 1). Indeed, from that volcano plot, only a couple of genes are DE according to DESeq (-log10 pval > 1, so a generous FDR cutoff of 10%), the others have decent fold changes but insignificant p-values which suggests low expression or high intra-species variance in expression (otherwise I would expect more signficant pvalues). What's going on here?

In light of this discordance it's very hard to interpret the rest of the box, which again would work better if integrated into the text as simply part of the results, and the more speculative sections starting in line 274 moved to the discussion. .

Lines 261, 265: Where is the data for these comparisons from? Is it all Kanton et al?

Line 294: I don't understand why these results are not considered significant given the reported pvalues? What exactly is being tested for here?

Line 323: Calling it polygenic selection feels a bit overenthusiastic, given the paucity of

Line 374: I do not understand why this was done. If degradation doesn't scale linearly with RIN, shouldn't including the whole value be more important? I understand the DE model will treat it as a linear factor and that can get murky, but in that case, more bins are perhaps a better solution here?

Line 396: Unclear if this means only samples > 10 reads/allele or only genes where all samples were >10 reads/allele were used for calculating the per gene ASE ratios.

Line 538: Surely this is methods?

Figure 2: It would be easier to see the results if each point had a much higher opacity, so readers could get an idea of density.

**Reviewer 2**

**Were you able to assess all statistics in the manuscript, including the appropriateness of statistical tests used? Yes: I was able to assess all statistical tests**

**Were you able to directly test the methods? No**

**Comments to author:**

In this paper, the authors follow up a simple but powerful idea to use allele-specific expression within species to better interpret allele-specific expression between species. They use published data and fairly straight-forward analyses to re-analyze and partly re-interpret human-chimpanzee differences. I find this really great: not only because it is an original idea that is backed up with relevant, very recent and appropriate data, but also because the paper is very clearly written. I think it could be almost published as is, but I would have two more major and a few trivial remarks

Major

1) Although I think I do understand that the normalization of the ASE data should take care of this, I am still unsure whether the mutational size of a gene (probably correlated with the size of a gene or the number/size of annotated cis-regulatory elements) has an influence on the population-scale ASE score, on the pHI score and hence on the entire analysis. I would find it interesting to test this in a similar manner as the other factors of concern (sex, tissue heterogeneity,..)

2) In the abstract it is claimed that the authors "identify signatures of lineage-specific selection on genes related to cellular proliferation, speech, and glucose metabolism". As especially the claim of selection on speech genes is a more bold one, I find the analysis not sufficient. I think the following would be needed:

a. The three GESA terms displayed in Figure 5 are just included in the list of gene sets identified at FDR<0.25. I find this cherry picking problematic (especially as I couldn't find Suppl. Table 3) and in general I find it helpful to rank the significant gene sets by effect size (i.e. enrichment) to judge how much cherry picking was involved

b. For the speech claim one would need to check additional datasets to support the finding from the hybrids in independent organoids (e.g. Kanton et al)

c. One would need to check whether the changes were actually on the human lineage (which would be required to connect them to speech), also using other datasets

d. Also an analysis of the cell types in which these genes are expressed and a bit longer discussion on how spastic dysarthia "may be connected to the human capacity for speech" (l. 310-311) would be needed (maybe another box?)

I think it is fine to make interesting speculations on phenotypic consequences in analyses like these, but I also find that bold claims shouldn't be made too easily. A cheap alternative to the analyses above would be to not make the speech claim so prominent, but it is a cheap alternative.

Minor remarks

1) I think describing the dataset used for population-level ASE (as in line 119 ff) already at line 104-105 would be better and less confusing

2) 1-2 sentence of explanation on the dataset that was used to construct the pHI score would be helpful

3) GSEAPY is first used as abbreviation only

4) Maybe use also a few more words to explain the sign test (in essence its like a test for a biased coin, no?)

5) Formatting error in Ref. 7 that was not published in 1979

**Authors Response**

**Point-by-point responses to the reviewers’ comments:**

**Reviewer 1**:

The manuscript by Starr et al advocates for the wider use of existing public data of within-species allele specific expression variation to calibrate inter-species differences and identify those that are likely to be of evolutionary interest from those that aren't. This is an approach that has long been theoretically advocated for in DE testing, as the references in line 74 prove, but hasn't been actively implemented in ASE testing. The manuscript has two parts, first laying out the idea behind the new method and its implementation, and then combines it with the sign test in an attempt to identify instances of concerted changes in gene expression between humans and chimpanzees that span multiple genes.

I do like the idea in principle, and I think it's a nice complement to existing challenges in inter-species evaluation of ASE differences, although suitable datasets will be hard to come by, sadly! Having said that, I also think that there's a lot of ideas here about the validity of sorting genes by p-value and then by p-value again that need more thoughtful exploring from a statistical standpoint, to validate the method; I write this review from the perspective of a potential user, which is what I am, not a statistician. I do highlight some places where the authors can begin addressing that in my more specific comments, as follows:

*Author Response: We thank the reviewer for pointing this out and agree that the sorting “by p-value and then by p-value again” is problematic. We have amended the method so that we only sort by the Mann-Whitney U p-value and all results now reflect this. Notably, this led to the additional identification of lineage specific selection on primary cilium assembly which we highlight in the manuscript. This also removes any concerns regarding statistical independence of the two ranking methods.*

Line 108: "We then use those p-values to separately rank genes with DESeq2 false discovery rate (FDR) less than or equal to 0.1 and greater than 0.1 such that genes with FDR less than or equal to 0.1 are always ranked higher than genes with FDR greater than 0.1." Isn't this sentence backwards? I presume the authors mean that they use the FDR to re-sort the list of p-values from their M-WU test such that MW-U p 0.15 and DESeq FDR 0.09 comes before MW-U p 0.00000000232 and DESeq FDR 0.101? This is also what is suggested by lines 419 onwards.

*Author Response: We agree these lines were confusing. As we have amended the method to no longer sort by DESeq2 FDR, we have removed these lines.*

Line 119: How far down the evolutionary tree do the authors think human data could be use to inform inter-species ASE comparisons?

*Author Response: We are generally not sure how divergent two species would have to be to largely alter the dosage sensitivity of many genes. We think that human ASE variance may be a useful proxy for other great apes or old-world monkeys, including hybrids between different species of old-world monkey. Augmentation of the human ASE distribution with ASE measured in other old-world primates may also improve comparisons across larger evolutionary distances. We have added the following sentences to address this: “In addition, while it is unclear if our method is applicable to hybrids between more diverged species than humans and chimpanzees, it is likely that ASE variance within one species will still be meaningful” (lines 361-363).*

Line 132: It's not clear to me that haploinsufficiency and constraint on ASE would go hand in hand. The assumption seems sensible on first pass, but simply considering ASE doesn't actually control for differences in expression, as they themselves state, and despite the correlation in the text it's hard to see the trend given that the authors have plotted the data as boxplots instead of eg a scatter plot or a 2D density plot or a hex bin plot would be preferable. It's also not clear why the data is binned in the plot for pHI but, presumably, not for the calculating of the correlation reported in text.

*Author Response: We appreciate the reviewer pointing out the potential disconnect between haploinsufficiency and constraint ASE and agree that there may not be a perfect relationship between the two. Our thinking is that in general, we expect that genes that are more likely to be haploinsufficient are likely to be more sensitive to decreases/increases in expression than genes that are not likely to be haploinsufficient. This is supported by the strong correlation between the probability of haploinsufficiency (pHI) and the probability of triplosensitivity found by the authors of the paper that introduces pHI. Overall, we think this distinction strengthens our result as even with perfect measurements the correlation would likely still be less than 1 due to these differences and we see a significant correlation despite these differences. To address concerns that expression level may be confounding these results, we have included an additional analysis in which we control for expression by pairing genes with*

*low and high pHI that have similar expression levels in GTEx. Despite the confounds this introduces that may weaken the relationship between pHI and ASE variance (for example, many haploinsufficient genes are only expressed at high levels in the brain and would have low average TPM across all of GTEx) we still observe a strong relationship between pHI and ASE variance. This is reflected in lines 138-140:*

*“Furthermore, genes with high pHI (> 0.75) value have lower ASE variance than genes*

*with low pHI (< 0.25) when controlling for expression (p < 10-150, paired t-test, Supp*

*Fig 2)”.*

*We binned pHI since its distribution is, unlike the GTEx ASE variances, bimodal and concentrated in the windows 0.8-1 and 0.2-0.4. Due to this, density plots and hexplots are difficult to interpret. As a result of this, in addition to the original binning strategy we have included a density plot of the data as a second panel in Supplementary Figure 1. We have also done our best to make the bins as informative as possible by:*

*1. Adding an explanation for why we binned the plot in the figure legend as well as clarified that no binning was done for computing the Spearman correlation. The explanation reads: “We binned pHI scores to more accurately represent the strength of the trend we observed as the non-uniform distribution of pHI scores combined with the nearly normal distribution of GTEx ASE variances makes it difficult to plot the correlation in an informative manner without binning” (lines 619-622).*

*2. Switching to violin plots as they are more informative of the distribution in each bin, and labeling the plot with the number of genes in each bin.*

Line 158: The fetal cortex ASE dataset is built from a max of 160 samples (as far as I can tell from looking at the reference - 85 neural progenitors and 74 differentiated progeny, although methods state 235 samples - where are the extra ones coming from?), a *lot* fewer than the GTEx dataset. Having said that, a visual count of the bar heights suggests that figure S2E included only ~70-80 data points. Are the CUX1 ASE values also outliers if GTEx is downsampled to the same number of samples? More broadly, and thinking of moving beyond human/ape comparisons - do the authors have any thoughts as to what is the minimum sample size needed to build a robust within-species ASE dataset? Does this vary between genes, eg, power estimates?

*Author Response: We have added an explanation to the Methods as to how the fetal cortex ASE dataset was processed to clarify this point. To summarize: 235 samples were collected. These samples were collected from 85 individuals for neurons and 74 individuals for neural progenitors, as well as fetal cortical wall from many of the same individuals. We summed reads for all samples from the same individual to more accurately approximate cortical spheroids which are a mixture of neural progenitors, neurons, as well as lower numbers of other cell types.*

*We also looked into how often the magnitude of the CUX1 GTEx ASE values are greater than the CUX1 human-chimpanzee ASE value. We found that the magnitude of a GTEx ASE value was greater than the magnitude of a CUX1 ASE value around 2.5% of the time. We have updated the manuscript to include this: “Furthermore, the persample ASE ratios are well outside the human fetal cortex population ASE distribution and only 2.5% of GTEx samples have a more extreme ASE value” (lines 212-214).*

*We agree with the reviewer that an analysis trying to understand the minimum sample size needed to use our technique would be broadly useful. To address this, we added a supplemental figure in which we down-sample GTEx to 5000, 4000, 3000, 2000, 1000, 500, 100, 50, 25, and 10 samples to understand how stable the ASE variances are at low sample sizes. This demonstrated that the ASE variances we compute are meaningful even with only 10 samples (see Supp. Fig. 3 in the revised manuscript).*

*Based on this, we think that our method is potentially applicable to most model and several non-model organisms with currently available datasets, although uniform processing of a few publicly available datasets may be required.*

Line 172: One thing missing for me from these analyses, and this section, is the actual rank correlation between rankings between those two methods, and the actual plotting of rank changes. By using the method in figure 1A, the naive experimenter would sort their genes by their between species ASE p-value only before GSEA, whereas with this method the ranking depends on two different metrics now - the difference in within and between species ASE *and* DE testing results, which if I understood line 108 correctly, will rearrange the gene list such that all DE genes are ranked higher than all non DE genes, but not change rankings within those groups?

So, how significant an impact does this process have, on average? What's the rank correlation between looking only at ASE and looking at ASE and DE? How much of a difference does not controlling for DE have? In figure 3, the authors skip straight to showing that genes that change rank a lot between the two approaches are enriched for neural development terms when considering data from hybric cortical spheroids. I note that according to line 428, this comparison is not directly performed at any point? Instead the authors compare the GO results between using only DE FDR to rank genes, and their combined method, rather than ASE pval, which seems far more relevant to the question (since DE methods actively control for intra-group variance using empirical Bayes approaches etc, but the M-WU approach does not, since each test is independent of all other tests and does not borrow data across genes or perform any sort of shrinkage)

*Author Response: We agree with the reviewer that this double ranking was confusing and ultimately unnecessary. Removing this addresses many of the points made here. However, we agree with the reviewer that additional analysis would still be helpful and thank them for pointing this out. In our view, there are three intuitive ways to rank the genes. First, one could follow the traditional route and rank by FDR from DESeq2 (or whichever DE testing program is used). Second, one could instead rank by the computed DESeq2 log2 fold-change. Since it is not standard to rank by fold-change we primarily focused on comparing to the DESeq2 FDR. The third option is to use our new method. All three options are highly correlated (Spearman’s rho ranging from 0.87 to 0.93) which is expected as the human-chimpanzee differences tend to outweigh differences in ASE variance. We agree that a visualization of how the ranks change between the three methods would be useful for readers of our paper and have included this as Supplemental Figure 4.*

Box 1: The format of the manuscript is confusing - results are shunted to boxes, where they're freely mixed with some fairly speculative discussion. I presume this is a carryover of formatting for a previous journal, but if not, I urge the authors to reconsider it, I found it frustrating. The CUX1 box in particular segues into what's clearly discussion after a brief walk through the results, and makes some fairly large speculative leaps with regards to neoteny that don't really belong in the middle of the results section. If the authors want to look more into it, there are brain organoid datasets out there from human and chimp.

*Author Response: We have removed the boxes and moved the more speculative parts to the Discussion.*

Line 250: "EDN1 had the strongest chimpanzee bias (mean log 2fold-change = -0.92, FDR < 0.025 at Day 150, Fig 4B)." I think there's something weird going on with figure 4B. EDN1 does indeed have a log FC of ~ -0.8 (could well be 0.92 but there's no tickmarks on the axes, please add those back) but the DE testing FDR-adjusted p value is 0.1 according to the y axis, not < 0.025 (-log10 (0.1) = 1). Indeed, from that volcano plot, only a couple of genes are DE according to DESeq (-log10 pval > 1, so a generous FDR cutoff of 10%), the others have decent fold changes but insignificant p-values which suggests low expression or high intra-species variance in expression (otherwise I would expect more signficant pvalues). What's going on here?

In light of this discordance it's very hard to interpret the rest of the box, which again would work better if integrated into the text as simply part of the results, and the more speculative sections starting in line 274 moved to the discussion.

*Author Response: We agree that the low fold-changes and relatively high DESeq2 pvalues makes it difficult to interpret this as evidence of selection. As such, we have removed this aspect of the plot and removed this analysis from the manuscript. Instead, we focused on EDNRB which is very highly ranked by our method. In addition, we have moved the more speculative parts of our EDNRB section to the Discussion. Finally, we also highlight a different pathway (assembly of cilia) that was identified as putatively under selection after making the changes that Reviewer 1 recommended. Notably, this enrichment for ciliary genes is broadly consistent with existing literature as multiple publications have highlighted changes in the expression of ciliary genes (e.g. Gokhman et al. 2021, Suresh et al. 2022) as having potentially played a role in human evolution.*

Lines 261, 265: Where is the data for these comparisons from? Is it all Kanton et al?

*Author Response: We have updated the figure legends to include the sources of the data. The data were drawn from three different studies including Kanton et al.*

Line 294: I don't understand why these results are not considered significant given the reported pvalues? What exactly is being tested for here?

*Author Response: We thank the reviewer for pointing out this ambiguity and agree that this section was confusing. We did consider these to be significant and apologize that this was not clear in the original manuscript. We have revised it to more clearly outline how our method is combined with the sign test and what we consider to be significant.*

*The revised text reads: “To examine human- and chimpanzee-biased genes separately, we sorted the list so that highly ranked genes with human-biased expression are at the top of the list and highly ranked genes with chimpanzee-biased expression are at the bottom of the list. In effect, this results in a test for directionallybiased cis-regulatory divergence that exceeds the cis-regulatory variation among most human alleles present in the GTEx population. More concretely, we first run GSEAPY preranked on this gene list and take all gene sets with GSEAPY FDR < 0.25 (the cutoff suggested by the GSEA authors) [28]. We then use the rank cutoff identified by GSEAPY to count the number of genes that are human-biased and the number that are chimpanzee biased, excluding non-significant genes. We then apply the sign test (in the form of the binomial test) and consider any gene sets with binomial FDR less*

*than 0.05 to be putatively under selection” (lines 260-270).*

Line 323: Calling it polygenic selection feels a bit overenthusiastic, given the paucity of

*Author Response: We have amended our language by including the phrase “probable lineage specific selection” and changed “known” to “identified”. We agree that identification of polygenic selection is difficult. However, the sign test is a wellestablished tool with a rigorous underlying null model. The p-values/FDR for several categories (e.g. gluconeogenesis or nonmotile primary cilium assembly) are significant for both GSEAPY and the binomial test (< 0.05 and < 0.005 respectively) and contain a large proportion of the genes in this gene set and our data (~75%), providing strong evidence for selection. Furthermore, for the glycan degradation category, low expression of these genes in the brain is conserved in mice and chimpanzees, but their expression is strongly increased in our data from the human allele. Lastly, we dug deeper into the spastic dysarthria category on Reviewer 2’s suggestion and suggest that the enrichment is more likely due to selection to maintain neuronal homeostasis rather than being directly related to speech. Overall, we think this constitutes strong evidence of polygenic selection on these genes.*

Line 374: I do not understand why this was done. If degradation doesn't scale linearly with RIN, shouldn't including the whole value be more important? I understand the DE model will treat it as a linear factor and that can get murky, but in that case, more bins are perhaps a better solution here?

*Author Response: We agree that running the model using more bins as well as our two-bin strategy would ensure that results from that dataset are robust. However, we realized when investigating this that none of the data shown or discussed was derived from the Khrameeva et al. dataset (it was a carry-over from the original version of the paper). As such, we have removed this citation as well as the part of the Methods section pertinent to it.*

Line 396: Unclear if this means only samples > 10 reads/allele or only genes where all samples were >10 reads/allele were used for calculating the per gene ASE ratios.

*Author Response: We have clarified this in the text as follows: “For example, if a sample had greater than or equal to 10 counts from one allele but fewer than 10 from the other allele for gene A, this sample would be removed from the calculation for gene A. However, if that same sample had greater than or equal to 10 counts from each allele for gene B, that sample would be included for the calculation for gene B” (lines 424-428).*

Line 538: Surely this is methods?

*Author Response: We have moved this to the Methods.*

Figure 2: It would be easier to see the results if each point had a much higher opacity, so readers could get an idea of density.

*Author Response: We have changed the plots in Figure 2 to density plots to better display the structure underlying the correlations we observe in line with this suggestion.*

**Reviewer 2:**

In this paper, the authors follow up a simple but powerful idea to use allele-specific expression within species to better interpret allele-specific expression between species. They use published data and fairly straight-forward analyses to re-analyze and partly re-interpret human-chimpanzee differences. I find this really great: not only because it is an original idea that is backed up with relevant, very recent and appropriate data, but also because the paper is very clearly written. I think it could be almost published as is, but I would have two more major and a few trivial remarks

Major

1) Although I think I do understand that the normalization of the ASE data should take care of this, I am still unsure whether the mutational size of a gene (probably correlated with the size of a gene or the number/size of annotated cis-regulatory elements) has an influence on the population-scale ASE score, on the pHI score and hence on the entire analysis. I would find it interesting to test this in a similar manner as the other factors of concern (sex, tissue heterogeneity,..)

*Author Response: In accord with the reviewer’s suggestion, we used the number of annotated cis-regulatory elements (CREs, drawn from ENCODE) as a proxy for mutational target size and computed the Spearman correlation between number of CREs and ASE variance. We observe a very weak positive correlation (rho = 0.07). We also correlated this value with the pHI scores (flipped so that lower pHI scores represent stronger constraint) and found a slightly stronger correlation (rho = 0.15). However, as the reviewer points out, using ASE controls for mutational target size as the two alleles of the same gene inherently have nearly identical mutational target sizes. As a result, we opted to add a statement about mutational target size to our results section: “In addition, biological factors (such as mutational target size) are also controlled for by comparing the expression of two different alleles” (lines 125-126). Due to this, we believe that further analysis of the relationship between constraint on gene expression and mutational target size, while very interesting, is outside the scope of our manuscript.*

2) In the abstract it is claimed that the authors "identify signatures of lineage-specific selection on genes related to cellular proliferation, speech, and glucose metabolism". As especially the claim of selection on speech genes is a more bold one, I find the analysis not sufficient. I think the following would be needed:

a. The three GESA terms displayed in Figure 5 are just included in the list of gene sets identified at FDR<0.25. I find this cherry picking problematic (especially as I couldn't find Suppl. Table 3) and in general I find it helpful to rank the significant gene sets by effect size (i.e. enrichment) to judge how much cherry picking was involved.

*Author Response: We thank the reviewer for pointing out this error; we intended that to say Additional File 2 rather than Additional File 3. Due to other changes to the manuscript it is now Additional File 1. In general, we consider the proportion of genes that agree in directionality to be the effect size of interest, but also take into account the normalized enrichment score from GSEAPY as well as the binomial p-value. Ranking by the proportion of genes with concordant ASE directionality, all four terms we highlight are in the top 10 terms identified by our method.*

*Ontology TermGSEAPY NES (Normalized Enrichment Score)GSEAPY FDRFDR Sign TestBinomial Effect Size*

b. For the speech claim one would need to check additional datasets to support the finding from the hybrids in independent organoids (e.g. Kanton et al)

c. One would need to check whether the changes were actually on the human lineage (which would be required to connect them to speech), also using other datasets

d. Also an analysis of the cell types in which these genes are expressed and a bit longer discussion on how spastic dysarthia "may be connected to the human capacity for speech" (l. 310-311) would be needed (maybe another box?). Analysis of which cell types they are expressed in also with the Kanton et al. I think it is fine to make interesting speculations on phenotypic consequences in analyses like these, but I also find that bold claims shouldn't be made too easily. A cheap alternative to the analyses above would be to not make the speech claim so prominent, but it is a cheap alternative.

*Author Response: Since these three points are interrelated and inform each other, we will address them together. We thank the reviewer for encouraging a deeper look at the “Spastic dysarthria” enrichment, and we agree that data from additional studies would strengthen our claim. To attempt to do this, we analyzed the data from Kanton et al. as well as data from additional studies including Pollen et al. 2019, Khrameeva et al. 2020, Sousa et al. 2017, and the recently published single nucleus RNA sequencing in Ma et al. 2022. However, potentially due to some combination of the relatively small fold-changes for these genes in the hybrids, factors that ASE controls for but traditional analysis of differential expression does not, and trans divergence between species, this analysis was inconclusive. As a result, we did not include results from this analysis in the updated manuscript.*

*With that said, we have added several sentences throughout the manuscript to clarify what we believe this signal of selection represents. We will expand further on these points here. Spastic dysarthria occurs as a result of selective degeneration of the axons and/or cell bodies of upper motor neurons that are exceptionally large and project axons across very long distances. These neurons are thought to be particularly vulnerable to degeneration. Notably, as there is not selective degradation of speech related motor neurons, spastic dysarthria is usually part of a constellation of symptoms that includes spastic ataxia. The genes underlying the enrichment for Spastic Dysarthria are involved in maintaining neuronal (and more specifically axonal) homeostasis. Rather than supporting speech per se, we think that these gene expression changes provide support for vulnerable neuron populations that may be even more vulnerable in humans than other species (or potentially in chimpanzees; as the reviewer points out, we cannot determine which lineage these changes occurred on without more data). As a result, we agree that we cannot say whether there is a direct connection to speech. Overall, while we think that the sign test provides evidence of selection without analysis of non-hybrid samples, we agree that claiming selection on the expression of genes related to speech is too strong given the evidence we were able to gather. As such, we have amended the manuscript in the following ways:*

*1.We have removed the mention of speech from the abstract and introduction.*

*2.We have clarified that these expression changes might support vulnerable upper motor neurons without a direct connection to speech. “Spastic dysarthria is a condition in which patients speak in a characteristic slow, regular, monotone manner as the result of the degeneration of vulnerable upper motor neurons [44]. Spastic dysarthria typically co-occurs with other symptoms such as spastic ataxia so, rather than being connected to speech, this finding may be connected to selection for maintenance of neuronal homeostasis [45]” (lines 300-304).*

*3.We have added a sentence stating that analysis of additional species will be needed to fully validate our findings. This sentence is: “Future work using outgroup species such as gorilla would be required to determine whether this selection occurred on the human or the chimpanzee lineage” (lines 319-321).*

Minor remarks

1) I think describing the dataset used for population-level ASE (as in line 119 ff) already at line 104-105 would be better and less confusing

2) 1-2 sentence of explanation on the dataset that was used to construct the pHI score would be helpful

3) GSEAPY is first used as abbreviation only

4) Maybe use also a few more words to explain the sign test (in essence its like a test for a biased coin, no?)

5) Formatting error in Ref. 7 that was not published in 1979

*Author Response: We thank the reviewer for these helpful clarifications and have addressed them as follows:*

*1) We have moved the following sentence up to lines 106-108 to describe the dataset used for population-level ASE sooner: “We use a large publicly available data set of post-mortem human RNA sequencing data, GTEx, to estimate ASE variance for every gene.”*

*2) We have included a sentence explaining the pHI score as follows: “The pHI score was developed by using statistical learning approaches on a massive dataset of disease-associated copy number variation in humans to predict the probability that each gene in the genome is haploinsufficient [24]” (lines 131-133).*

*3) We have spelled out GSEAPY as “Gene Set Enrichment Analysis in Python” in our first use (line 192).*

*4) We have added the following sentence to our explanation of the sign test: “This is similar in principle to testing whether a coin is biased based on the outcome of multiple coin flips.” (lines 240-241).*

*5) We have fixed this formatting error to reflect the actual publication data, (lines 699, 705, 748, 800, 812).*

**Second round of review**

**Reviewer 1**

Thanks to the authors for the changes made in this version of the manuscript. It's great to see that most of the results were not dependent on the double-ranking method. I find the authors have addressed my comments satisfactorily, and all I have to point out is something that I think is probably a typo on line 432: "(i.e. mean of human and chimpanzee read count less than 25 and mean of chimpanzee read count less than 25)." Pretty sure the 'and chimpanzee' isn't needed!

**Reviewer 2**

Thanks for these clear, constructive and helpful revisions. All my concerns have been addressed sufficiently.
